# Types of therapeutic errors in the management of osteoporosis made by physicians and medical students

**DOI:** 10.1186/s12909-022-03384-w

**Published:** 2022-04-27

**Authors:** Olivia Tausendfreund, Leah T. Braun, Ralf Schmidmaier

**Affiliations:** Medizinische Klinik und Poliklinik IV, LMU Klinikum, Ziemssenstr. 5, 80336 Munich, Germany

**Keywords:** Therapeutic errors, Clinical reasoning, Diagnostic errors, Medical education

## Abstract

**Background:**

Clinical reasoning is of high importance in clinical practice and thus in medical education research. Regarding the clinical reasoning process, the focus has primarily been on diagnostic reasoning and diagnostic errors, but little research has been done on the subsequent management reasoning process, although the therapeutic decision-making process is at least equally important. The aim of this study was to investigate the frequency of therapeutic decision errors and the cognitive factors leading to these errors in the context of osteoporosis, as it is known to be frequently associated with inadequate treatment decisions in clinical practice worldwide.

**Methods:**

In 2019, 19 medical students and—for comparison—23 physicians worked on ten patient cases with the medical encounter of osteoporosis. A total of 254 cases were processed. The therapeutic decision errors were quantitatively measured, and the participants’ cognitive contributions to therapeutic errors and their clinical consequences were qualitatively analysed.

**Results:**

In 26% of the cases, all treatment decisions were correct. In the remaining 74% cases, multiple errors occurred; on average, 3 errors occurred per case. These 644 errors were further classified regarding the cognitive contributions to the error. The most common cognitive contributions that led to errors were faulty context generation and interpretation (57% of students, 57% of physicians) and faulty knowledge (38% of students, 35% of physicians). Errors made due to faulty metacognition (5% of students, 8% of physicians) were less common. Consequences of these errors were false therapy (37% of cases), undertreatment (30% of cases) or overtreatment (2.5% of cases).

**Conclusion:**

The study is the first to show that errors in therapy decisions can be distinguished and classified, similar to the already known classification for errors in diagnostic reasoning. Not only the correct diagnosis, but particularly the correct therapy, is critical for the outcome of a patient.

**Supplementary Information:**

The online version contains supplementary material available at 10.1186/s12909-022-03384-w.

## Background

### Errors in medicine

Errors in medicine are frequent and might endanger patients’ safety. Approximately, every 10th diagnosis is wrong [1]. Also, it is already known that there are a high number of treatment errors in clinical practice [2]. For example, the number of treatment errors in Germany in a single year is about 40,000 [3]. Errors occur in atypical cases, in rare diseases or in patients with uncommon course of a disease, but they also occur in common, interdisciplinary treated diseases that affect millions of people each year. One example for that is the medical encounter of osteoporosis: Despite the high prevalence, especially among elderly individuals in many European countries, for example, in Germany (29.2%), France (29.6%) or Italy (30.1%), the medical care of those affected is still in urgent need of improvement [4], although treatment indications and choices for a specific treatment are quite standardized by evidence-based clinical guidelines [5]. The selection of the correct therapy is crucial for the outcome of a patient. Errors in the diagnosis and treatment of osteoporosis lead to high economic costs, patients might fall into social isolation due to immobilisation and thus, they might face a loss of quality of life. As the danger of errors in medicine is not negotiable, in the past decades, medical education focused more and more on clinical reasoning and errors.

### Management reasoning and cognitive errors in therapeutic decision making

Clinical reasoning includes not only diagnostic reasoning but also management reasoning [6]. It summarises the entire process from considering a diagnosis, initiation of appropriate examinations, stating a final diagnosis, making therapeutic decisions and monitoring the therapy. Quite recently, David Cook and colleagues stressed the importance of so-called management reasoning and the fact that only very limited scientific data is available [7, 8]. In several aspects, management reasoning seems to be even more complex than diagnostic decision-making [7, 8], as it includes ongoing monitoring of a patient or shared decision-making with the patient [7, 8]. Therapeutic decision-making is an important part of management reasoning. Therapeutic decisions depend on several aspects (patient, resource availability, etc.) and sometimes it is very difficult to judge a therapeutic decision as “correct” or “best”, as pointed out by Cook et al. [7, 8]. Within the cognitive process of therapeutic decision-making, errors can occur. With the aim of optimizing processes in medicine further, a closer examination of management reasoning is therefore of increasing interest. So far only little research has been done in this important field.

### Classification of reasoning errors

Neither the frequency of errors in therapy decisions made by medical students nor the cognitive contributions to these errors have been studied systematically thus far. Knowledge about the frequency and nature of cognitive errors in a certain medical context is of high importance for the development of medical education strategies to overcome them and to avoid patient harm.

Although little is known about therapeutic errors themselves, a lot of studies focused on diagnostic reasoning and errors in the diagnostic process: Diagnostic errors are frequent—both in everyday clinical practice and in simulations—and can be classified [9–13]: In a study with 100 diagnostic cases by Graber et al. [9], a diagnosis was considered incorrect if it was a misdiagnosis (wrong diagnosis) or a missed diagnosis (too late or not at all). Data on 100 diagnoses made by doctors were collected and comprehensively evaluated. The cases were evaluated by a review of the medical record, interviews with involved practitioners and analysis of quality assurance activities. Graber et al. separated system-related errors from cognitive errors. System-related errors occur due to organizational, communication, policy and procedural problems and need to be addressed within the given organizational context. Cognitive contributions to errors could be further subdivided into “faulty knowledge”, “faulty data collection”, “faulty synthesis – faulty information processing” and “faulty synthesis – faulty verification”. To obtain the exact origin of the errors, 25 subcategories have been introduced, such as “insufficient or defective skills” and “overestimation or underestimation of a symptom or a finding” [9]. Most cases were assigned to more than one category, as many diagnostic errors were multifactorial.

### Adaptation of the diagnostic error classification into therapeutic error classification

As with Graber’s categorization an already comprehensive classification system for diagnostic errors exists, the classification was adopted to therapeutic decisions. First of all, all categories for cognitive contributions to errors as named by Graber were listed. In a second step, all categories that were not applicable to an online study environment or treatment situation were deleted. Examples for these deleted categories are: “failure to screen”, “misidentification of a symptom”, or “distraction by other goals or issues”. A full list of all omitted categories is shown in the supplement. Finally, 5 of Graber’s categories were integrated into the therapeutic error classification but further subdivided to specify them. For example, Grabers category “Knowledge base inadequate or defective” was further divided into three categories: “Lack of knowledge of a necessary therapeutic action”, “Lack of knowledge of a special indication”, “Lack of knowledge of contraindication”. In Table 1, the final therapeutic error classification in comparison to Graber’s classification is shown. For each category, a definition and an example is given.

**Table 1:** Classification of cognitive contributions to errors

Graber et al. [9]	Definition	Example	Tausendfreund et al.	Definition	Example
Type			Type		
**a. Faulty knowledge**			**a. Faulty knowledge**		
*Knowledge base inadequate or defective*	*Insufficient Knowledge of relevant condition*	*Providers not aware of fournier gangrene*	*Lack of knowledge of a necessary therapeutic action*	*Clinician has insufficient knowledge of all therapeutic steps*	*Basis medication is incomplete or completely forgotten*
			*Lack of knowledge of a special indication*	*Clinician has insufficient knowledge of a special indication for a specific therapeutic action*	*Patient with reoccurring vertebral body fractures receives alendronate (teriparatide would be indicated)*
			*Lack of knowledge of contraindications*	*Clinician has insufficient knowledge of all contraindications*	*Patient with severe kidney failure is prescribed a bisphosphonate therapy*
**b. Faulty Synthesis: Faulty information processing**			**b. Faulty context generation and interpretation**		
*Faulty context generation*	*Lack of awareness/consideration of aspects of patient’s situation that are relevant to diagnosis*	*Missed perforated ulcer in a patient presenting with chest pain and laboratory evidence of myocardial infarction*	*Misidentification of information as a contraindication*	*Clinician identifies given information as faulty as a contraindication*	*Wrong contraindications are stated (young age, certain medication, male sex)*
			*Failure in recognizing contraindications*	*Clinician fails to identify information as a contraindication*	*Female patient with risk for thrombosis receives estrogen*
*Overestimating or underestimating usefulness or salience of a finding*	*Clinician is aware of symptom but either focuses too closely on it to the exclusion of others or fails to appreciate its relevance*	*Wrong diagnosis of sepsis in a patient with stable leukocytosis in the setting of myelodysplastic syndrome*	*Underestimation of a finding in the process of considering patients` individual risk*	*Deficiency in interpreting the patient’s individual 10-year -fracture-risk, leading to an underestimation*	*Advanced patient age or female sex is overlooked; the T-score is miscalculated*
			*Faulty interpretation of results resulting in “undertreatment”*	*Clinician interprets given information as faulty, resulting in too little of an amount of therapy for the patient*	*post-menopausal condition is overlooked*
			*Overestimation of a finding in the process of considering patients’ individual risks*	*Deficiency in interpreting the patient’s individual 10-year-fracture-risk, leading to an overestimation*	*BMI is misjudged; the T-score is miscalculated*
			*Faulty interpretation of results resulting in “overtreatment”*	*Clinician interprets given information as faulty, resulting in too much of an amount of therapy for the patient*	*Fractures on non-osteoporosis relevant party of the body are included in risk calculation (*e.g.*, rib, toe, …)*
			*Failure to leave the common path of procedures*	*Clinician sticks to common therapy ignoring a special indication*	*In this case, the participant stated that ‘that the decision is based on personal experience’*
**c. Faulty synthesis: Faulty Verification**			**c. Faulty metacognition**		
*Premature closure*	*Failure to consider other possibilities once an initial diagnosis has been reached*	*Wrong diagnosis of musculoskeletal pain after a car crash: ruptured spleen ultimately found*	*Possible overconfidence*	*Clinician fails to question their own findings*	*Necessary additional consult with a specialist is not performed (patient case with severe mastocytosis)*
*Failure to consult*	*Appropriate expert is not contacted*	*Hyponatremia inappropriately ascribed to diuretics in a patient later found to have lung cancer; no consultations requested*			
			*Lack of confidence*	*Clinician fails to trust their own findings*	*Additional consult with a specialist with a low threshold*

### Research question

The aim of the study was to investigate the frequency and cognitive contributions to therapeutic errors in the context of osteoporosis made by medical students and physicians. We focused on therapeutic errors in the context of osteoporosis in this study, as it is a context in which therapeutic decisions can easily be rated as correct or incorrect.

## Methods

Medical students in the clinical phase of medical school as well as trained physicians who worked as primary care physicians or doctors of internal medicine were recruited for this study. Only medical students who completed their course of internal medicine, including the topic of osteoporosis, took part in the study. All participants worked on a management reasoning course in the electronic learning environment CASUS [14]. Participants received a standardized introduction and worked on standardized clinical cases. Participants’ answers (free texts and multiple questions answers) were electronically recorded.

Approval or the study was obtained from the Ethical Committee of the Medical Faculty of LMU Munich.

### Participants

Nineteen medical students provided answers for all 10 cases and all additional questions. A total of 190 patient cases were solved by them. The average age of this study group was 23.4 ± 1.4 years. Forty-seven percent of the participants were female, and 68% were in their 5th year of medical school at the time of the study. 23 doctors took part in the study as well and dealt with 64 cases. The doctors were, on average, 48 ± 9.6 years old and 93% of the participants in this study group were male. The students were given a financial incentive of 20 Euros for participating.

### Computer-based management reasoning course -

#### Study environment and pilot phase

The study was carried out in 2019 on the online learning platform CASUS at Ludwig Maximilian University [15]. The materials for the study were generated by one study author (OT) on the basis of the German osteoporosis guidelines of 2017 [16] and reviewed by two experts.

First, participants watched a 20-min-long video covering the established diagnostic steps in osteoporosis and the therapy algorithm including the basic drug and the specific drug therapy for osteoporosis. At the same time, the participants also received a short booklet with the most important facts. Secondly, participants answered a sociodemographic questionnaire.

**Fig. 1:**
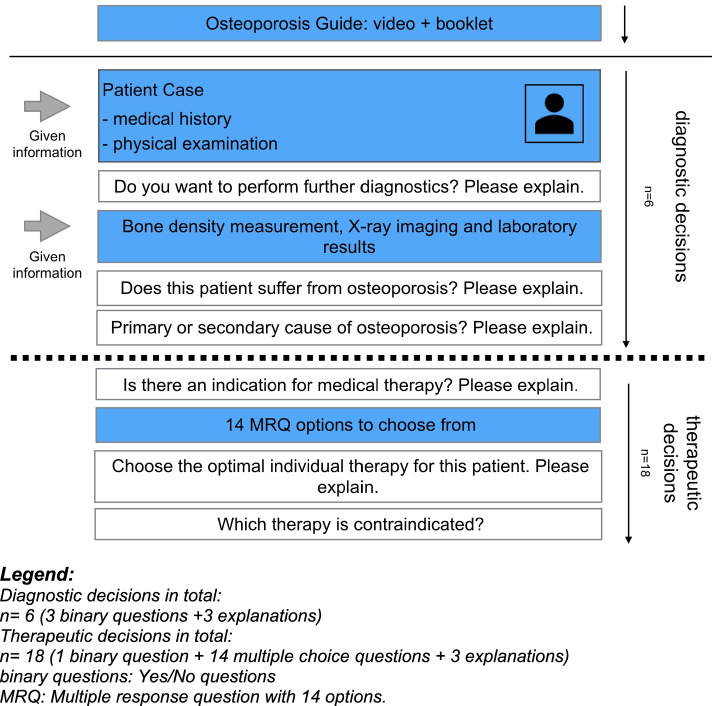
Case structure. Legend: Diagnostic decisions in total: *n* = 6 (3 binary questions + 3 explanations). Therapeutic decisions in total: *n* = 18 (1 binary question + 14 multiple choice questions + 3 explanations) binary questions: Yes/No questions. MRQ: Multiple response question with 14 options

Third, the participants worked on ten clinical case vignettes in the electronic learning platform CASUS. The structure of the cases is outlined in Fig. 1. The patients of these case vignettes suffered from different types of osteoporosis or osteopenia. The cases included a detailed medical history and the physical examination of a fictional patient. Bone density measurements, X-ray images and laboratory results were provided if the participant asked for them in the next step. An example patient case is given in the supplemental material. A pilot study was carried out with 4 medical students who did not participate in the actual study to assess and, where needed, improve the design of the study and the difficulty of the cases. All cases were solved in a reasonable amount of time, and mean difficulties were as expected without floor or ceiling effects. Incoherence and redundancies were removed and the layout was optimized.

### Assessment of management reasoning

**Fig. 2:**
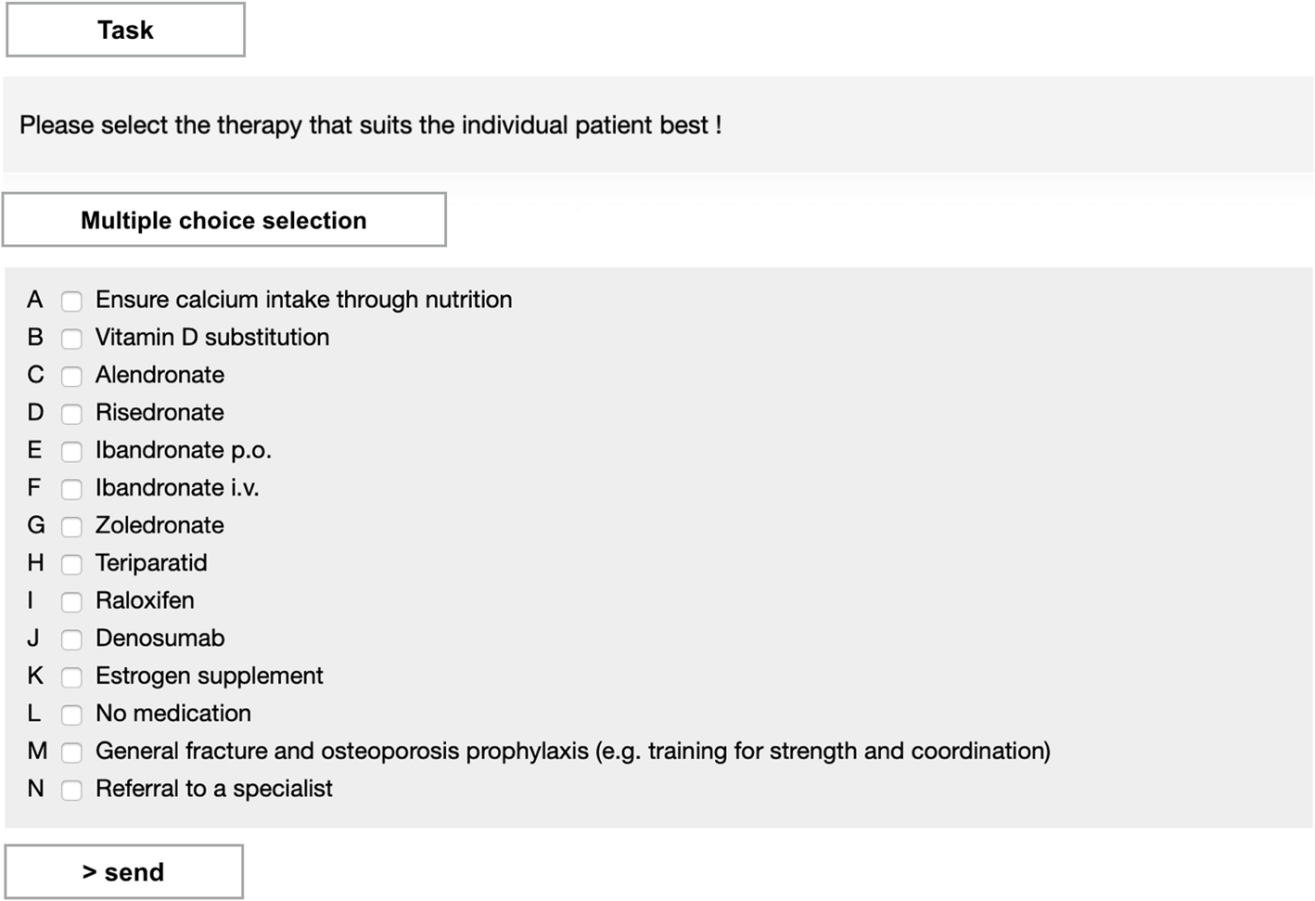
User Interface: Individual therapy selection

To complete a case, 3 multiple-choice questions about the diagnosis and therapy had to be answered and three times, participants had to explain their answers in free texts. These 6 tasks are also stated in Fig. 1 (three closed questions and three free texts). First, the participants had to decide after medical history and physical examinations, if they wanted to perform further diagnostics. Secondly, they had to state if the patient suffers from osteoporosis or not. If so, the cause of osteoporosis (primary or secondary) had to be stated. Participants had to explain their answers after each question (free text). This first part focused on diagnostic accuracy. In the second part, the participants had to choose a specific treatment for each individual case and – if there was an indication for treatment - justify their decisions. As shown in Fig. 2, participants could choose therapy from a list with 14 different options. The participants had to write down their reasons for a decision in a text box in the CASUS platform (free text).

### Quantitative and qualitative analysis and statistics

#### Quantitative analysis of error frequency

Following the diagnostic decision, three different types of therapeutic errors were assessed quantitatively (as a percent-correct score):

1) errors in making the decision to indicate a treatment;

2) errors regarding contraindications;

3) other errors in choosing the correct individual treatment.

The answers were binary coded as correct or incorrect according to an expert solution of the case. These aspects were solely assessed quantitatively as frequencies (for example, the frequency of errors to initiate a treatment).

#### Qualitative analysis of types of errors

To assess the cognitive contributions to errors, the modified taxonomy by Graber et al. was used as described above. To assign the errors to a category, the free texts of the students in which they explained their decisions were analysed. Also, the answers of the multiple questions were taken into account in which the participants chose a specific treatment. The free texts were read by one investigator and assigned to a matching category. For example: A participant prescribed oestrogen to treat a patient. This patient had a high risk for thrombosis due to the history. Therefore, oestrogens cannot be prescribed. This error was assigned to the category “failure in recognizing contraindications”. The same procedure was done with all other cases, in which the patient received a wrong or suboptimal therapy. One investigator analysed all free-texts, a second rater coded 10% of the free texts. The interrater coefficient analysed with Cohen’s kappa was k = 0.80.

#### Qualitative analysis of consequences of errors

Finally, the consequences considering current treatment guidelines and expert opinions of the errors for the patient were assigned to one of the following categories:

1) inadequate treatment choice;

2) overtreatment;

3) undertreatment;

4) no therapy (although this would have been necessary).

For the quantitative analysis, SPSS 26 was used. The participants’ diagnostic accuracy was quantitatively assessed, and their answers were binary coded as correct or incorrect according to a sample solution of the case.

## Results

### Frequency of diagnostic and therapeutic errors

**Fig. 3:**
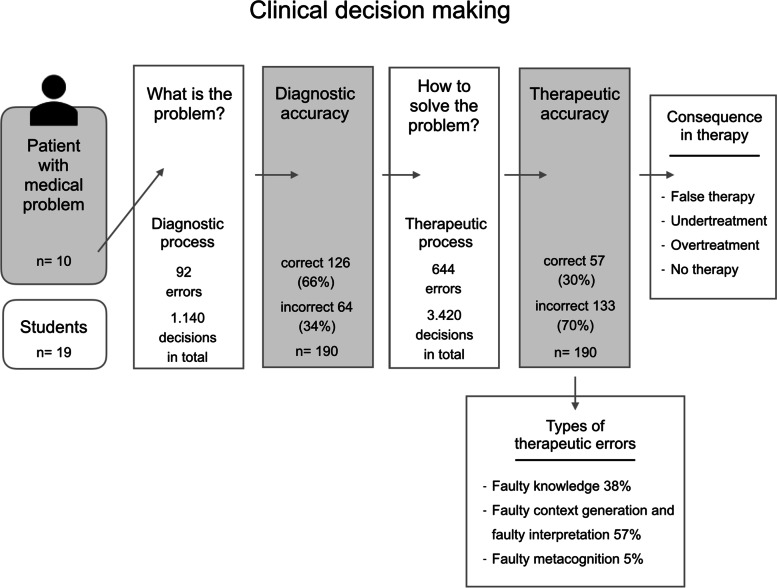
Overview: Therapeutic errors in clinical decision-making

The students misdiagnosed the patients in 64 cases ((34%); physicians: 21 cases (33%)). During this diagnostic process, 92 errors (physicians: 35 errors) were found in the 1.140 decisions that had to be made. These included, for example, the nonrecognition of osteoporosis or incorrect differentiation between primary and secondary osteoporosis (Fig. 3).

Despite a correct or incorrect diagnosis, we included all cases in the analysis of therapeutic errors, as a correct treatment was only partly dependent on diagnostic accuracy: in 52 of the 126 patient cases correctly diagnosed by students and 15 of the 43 cases correctly diagnosed by physicians, all treatment decisions were correct, whereas in 5 cases with incorrect diagnoses, the treatment decisions were correct (students) and 2 cases with incorrect diagnoses were treated correctly by the physicians. Overall, in 67 out of the 254 cases, indication for therapy and individual treatment selection was correct, which means that 26% of the cases handled by both study groups were correctly solved (27% students and 23% physicians). In 4% of the cases, the treatment choices were correct, while the indication was incorrect.

The students chose incorrect or incomplete treatment in 133 of the 190 (70%) cases. According to our study design (Fig. 1), three different therapeutic decisions needed to be made (indication, choice of treatment, contraindication). Of note, some students and physicians made errors regarding the indication, contraindication and choice of treatment; therefore, all in all, errors occurred statistically in more than 100% of cases. Approximately one-fifth of the students made errors regarding indications (21%), and 50% made errors regarding contraindications to treatment. The majority of faulty decisions were regarding individual therapy decisions (75%). Regarding the individual final therapy decision, 57 patients (30%) were correctly treated by the students, while 133 (70%) received incorrect or incomplete therapy. The more experienced physicians made the same number of errors regarding indications (18%) and contraindications (52%) and as many errors as the students regarding the individual therapy decisions (73%). Furthermore, regarding the final therapy decision 17 patients were correctly treated (27%), at the same time 47 patients (73%) received incorrect or incomplete therapy by the physicians.

### Cognitive contributions to therapeutic errors and consequences

Overall, the students made 644 errors during the therapeutic decision process (physicians, 223 errors), which included 3420 therapeutic decisions that had to be made. These errors were assigned to one or more of the categories shown in Table 2. All errors could be divided into three main categories: faulty knowledge (38%), faulty context generation and interpretation (57%), and faulty metacognition (5%) (Fig. 3, Table 2).

**Table 2:** Cognitive contributions to therapeutic errors

Type	Students’ therapeutic errors (***N*** = 644)	Physicians’ therapeutic errors (***N*** = 223)
**a. Faulty knowledge**	**247 (38%)**	**78 (35%)**
*Lack of knowledge of a necessary therapeutic action*	119	29
*Lack of knowledge of a special indication*	96	30
*Lack of knowledge of contraindications*	32	19
**b. Faulty context generation and interpretation**	**365 (57%)**	**128 (57%)**
*Misidentification of information as a contraindication*	39	16
*Failure in recognizing contraindications*	7	2
*Underestimation of a finding in the process of considering patients` individual risk*	100	43
*Faulty interpretation of results resulting in “undertreatment”*	155	48
*Overestimation of a finding in the process of considering patients’ individual risks*	30	7
*Faulty interpretation of results resulting in “overtreatment”*	34	9
*Failure to leave the common path of procedures*	0	3
**c. Faulty metacognition**	**32 (5%)**	**17 (8%)**
*Lack of confidence*	22	12
*Possible overconfidence*	10	5

Cognitive errors led to false or suboptimal therapy in 37% of the cases (71 cases) and to undertreatment or no therapy in 30% of the cases (57 cases). Overtreatment occurred seldomly, in only 2.5% of the cases (5 cases). However, in 50 out of 190 cases (26%), the students referred the patients to a specialist, although it would not have been necessary. In one of the cases, however, referring the patient to a specialist was indicated, but only 9 of the 19 students did so.

The physicians’ decisions led to false/suboptimal results in 42% of the cases (27 cases) and to undertreatment in 25% of the cases (16 cases). Overtreatment did not occur. Similar to the students, the physicians referred a patient to a specialist without indication in 30% of cases (Fig. 3).

## Discussion

### Summary and discussion of results

Comprehensive error analysis shows which errors can arise in treatment decision-making and on which cognitive contributions they are based. For this purpose, the diagnostic error classification according to Graber et al. [9] had to be slightly changed despite some similarities, i.e., certain cognitive contributions to the mistakes made in the therapy process differed from the cognitive contributions to errors in diagnostic decisions.

The therapy process always goes hand in hand with the diagnostic process. Right and wrong decisions are dependent on each other and have a direct influence. Therefore, a correct clinical decision always consists of two equally important parts: diagnostic accuracy and therapeutic accuracy. While diagnostic accuracy might have been correct, therapeutic errors could still occur. On the other hand, in some cases, a correct treatment decision was made despite diagnostic incorrectness. This phenomenon is already known from diagnostic accuracy but in an opposite manner: Sometimes, a false diagnostic process results in a correct diagnosis [17].

In general, both groups often succeeded in providing the correct therapy indication. The participants mostly performed well up to this point, but in the following step of choosing the correct therapy for the individual patient, the error counts greatly increased. This showed that both groups were able to recognize the need for therapy, but in the end, correct treatment often failed due to incorrect therapy selection from the given possibilities (Fig. 2).

As previously stated, both the students and doctors made similar errors with similar cognitive contributions to the errors. The fact that the doctors were just as bad or good as the students can be explained by content-specific diagnostic competence, which appears to be very similar. The most frequent error was faulty context generation and interpretation. This error increased relatively with clinical experience due to less faulty knowledge. In many circumstances, this content-specific competence is acquired while treating the first couple of patients. From a didactical point, providing a few training sessions with simulated patients is mandatory to ensure patient safety in clinical practice [18]. An incorrect selection did not necessarily mean that the therapy choice had a serious negative effect on the patient’s health in every case, and the choice of therapy was sometimes “only” not the best or slightly incomplete. This is in line with the definition of Cook et al. for classifying management plans as “more or less reasonable” [7, 8]—while diagnostic decisions can be relatively easily rated as “correct” or “incorrect”, there seems to be more of a variety in treatment decisions. A study on the distinction of how serious a mistake is has not been performed. This may explain the relatively large count of incorrect individual therapy selections in both groups. However, a treatment gap of up to 85% in osteoporosis patients is well known from the literature [19], which is in line with our results.

As stated by Cook et al. [7, 8], one major research topic is the understanding of cognitive processes in management reasoning. We have developed a case simulation platform for training for therapeutic decision-making in osteoporosis and have identified the most frequent cognitive errors. This provides the opportunity to further explore instructional support [20] in future studies, even in a collaborative setting [21]. Accordingly, we propose a concept of collaborative therapeutic reasoning.

An interesting but minor new cognitive contribution to errors that was primarily found in the doctors’ study group, was the fact that therapy was sometimes chosen based on habit rather than on evidence-based knowledge. The participants tended to select and give common drugs, even if another specific therapy would have been more suitable in the given case. This might be a misleading strategy to reduce cognitive dissonance [22].

In our study environment, a patient was at a slightly higher risk of receiving too little therapy than too much therapy. This might be characteristic of osteoporosis, as another study regarding prescribed antibiotics has shown: physicians often prescribe unnecessary antibiotics out of fear and the desire to quickly fix a problem [23]. Especially for older patients, unnecessary drug prescriptions can even endanger the health of a patient.

### Strengths and limitations

Nonetheless, this study also has some limitations, the most important being the content specificity. It is well known that clinical reasoning is case and content-specific. Our study only focused on the medical topic of osteoporosis; error frequency and cognitive contributions to errors might be completely different in other contents.

The monocentric design of the study might be a minor limitation. At other locations with different curricular structures and focuses, the results may differ. The study was performed with relatively small study groups. This might have given individual decisions more importance and could have influenced the results to an extent we cannot examine. Additionally, due to the small study groups, we did not perform an analysis on person-specific or case-specific errors. This is an important aspect for future studies. Due to the design of the study, we cannot comment on system- or patient-related factors that lead to therapeutic errors, as other studies have [23]. Another limitation of the study is the sex imbalance in the physicians’ group. The majority of participants in this group were male. An influence on the results by sex cannot be excluded. Most likely, this imbalance may be explained by real-world sex imbalances in Germany: 63% of medical students in the first semester are female, whereas 89% of leading physicians are male [24].

The study has important strengths. To our knowledge, this is one of the first studies to analyse the therapeutic errors made by medical students. Common realistic patient cases, which can be found in everyday clinical practice, were used for this purpose. In a comment by Cook et al. [7], different research priorities were named, including a better understanding of management errors. Our study complements the current literature.

## Conclusions and outlook

In this study, errors made in the therapy decision were systematically analysed, both in a group of students and a group of doctors. Most errors were made due to inconsistent interpretation. This emphasizes that not only the transmission of knowledge per se but also the ability to logically interpret information seems to require more attention, regardless of one’s individual expertise in terms of years practising medicine.

We were able to show that osteoporosis is seemingly underestimated and because of the wide diversification, a highly difficult topic both in diagnosis and therapy. This needs to gain attention in the curriculum and training of young novices. Closing these knowledge gaps could have a strong positive impact, taking the growing prevalence and social relevance of this disease into account.

This is highly important to impart the skills required for independent ‘clinical reasoning’ and to prevent errors that put patients’ well-being at risk.

## Supplementary Information

**Additional file 1.**

## Data Availability

The datasets analysed during the current study are not publicly available as the original data set is in German but the datasets are available from the corresponding author on reasonable request.
