# Supplementary material for: Types of therapeutic errors in the management of osteoporosis made by physicians and medical students
Source: BMC Med Educ. 2022 Apr 27;22:323. doi: 10.1186/s12909-022-03384-w (PMC9044589; doi:10.1186/s12909-022-03384-w)
Supplement: Supplementary file 1 — Additional file 1. [file 12909_2022_3384_MOESM1_ESM.docx]

**Supplement**

**List of omitted categories of Graber’s taxonomy**

| - Faulty test or procedure techniques - Failure to screen (prehypothesis) - Poor etiquette leading to poor data quality - Faulty triggering - Misidentification of a symptom or a sign - Distraction by other goals or issues - Faulty interpretation of a test result - Reporting or remembering findings not gathered - Failure to periodically review the situation - Failure to gather other useful information to verify diagnosis - Overreliance on someone else`s findings or opinion - Failure to validate findings with patient - Confirmation bias - Skills inadequate or defective - Ineffective, incomplete or faulty work up - Ineffective, incomplete or faulty history and physical examination - Faulty detection or perception - Failed heuristics - Failure to act sooner - Failure to order or follow up on appropriate test |
| --- |

**Case example**

Your next patient is M. Messner, 61 years old.

**Medical history**
He complains about a decrease in general performance. In addition, persistent muscle and knee pain have made life a lot more difficult for him. He states that he was always athletic and in fact, in excellent health. In recent times, however, he has been unable to keep up with his hiking group due to pain. This is similar to a situation 5 years ago when his meniscus was torn while skiing, which was then conservatively treated. Since his youth, he has suffered from asthma, which he always has well under control with retard tablets and an inhaler. However, lately, the air shortage increased significantly, so 4 months ago, he also started cortisone therapy. The patient denied nicotine and alcohol consumption, and allergies were not known.

**Past medical history**: Bronchial asthma

**Medication**: Oral glucocorticoid therapy over 4 months (8mg prednisolone equivalent /day), additionally a beta-2-mimetikum.

**Physical examination**

Awake, 4 times-oriented patient in average general condition and a normal nutritional state (1.69 m, 63 kg, BMI 22.1).
Vital parameters: blood pressure 130/90 mmHg, heart rate 70/min, respiratory rate 15/min, temperature 36.8 degrees
Cardiovascular system: normal and rhythmical heart sounds, no pathological heart sounds, no flow of noise. No jugular vein distention, peripheral oedema, peripheral pulses (A. radialis bds., A. dorsalis pedis bds., A. tibialis posterior bds.) strongly palpable.
Respiratory system: No chest wall deformities, no lip cyanosis, no dullness on percussion, and lung borders on both sides were displaceable by 2 finger widths upon breathing. Vesicular breath sounds. No damp or dry breathing noises, no wheezing or stridor. Breast spine was not painful.
Abdomen: Inspection was inconspicuous. Vivid intestinal sounds over all four quadrants, with no abdominal flow noise. Abdominal wall was soft, palpation was not painful, with no palpable resistance. Liver with a soft edge was palpable without pain. The spleen was not palpable. No hernias. No kidney-percussion. Lumbar spine was not painful under percussion.
Neck: no pathologically enlarged cervical lymph nodes were palpable, the thyroid was soft, and no knots were palpable.
